# Efficacy of Danlou tablets in patients with acute coronary syndrome undergoing percutaneous coronary intervention: a multicenter prospective cohort study

**DOI:** 10.3389/fcvm.2024.1420194

**Published:** 2024-09-24

**Authors:** Yajie Cai, Qiaoning Yang, Ruixi Xi, Furong Yang, Feng Gu, Yang Zhao, Ming Guo, Guoju Dong, Zhuye Gao, Changgeng Fu, Peili Wang, Jianpeng Du, Dawu Zhang, Wenhui Duan, Lizhi Li, Dazhuo Shi, Ruina Bai

**Affiliations:** ^1^ Xiyuan Hospital, China Academy of Chinese Medical Sciences, Beijing, China; ^2^ NMPA Key Laboratory for Clinical Research and Evaluation of Traditional Chinese Medicine, Xiyuan Hospital, China Academy of Chinese Medical Sciences, Beijing, China; ^3^ National Clinical Research Center for Chinese Medicine Cardiology, Xiyuan Hospital, China Academy of Chinese Medical Sciences, Beijing, China

**Keywords:** Danlou tablet, percutaneous coronary intervention, acute coronary syndrome, efficacy, Chinese medicine

## Abstract

**Background:**

Danlou tablets (DLTs) have been widely used to treat coronary heart disease in China. However, the benefits associated with DLT for patients with acute coronary syndrome (ACS) undergoing percutaneous coronary intervention (PCI) in routine practice require further investigation.

**Purpose:**

To investigate the effectiveness of DLT in patients with ACS undergoing PCI.

**Methods:**

This multicenter prospective cohort study for patients with ACS undergoing PCI was conducted in 40 centers in mainland China from February 2012 to December 2018. This trial is registered under ChiCTR-OOC-14005552. Patients were assigned to either the DLT group or the conventional medicine (CM) group based on whether they used DLT prior to enrollment. The duration of DLT use (1.5 g, three times a day) was 12 months. The primary endpoint comprised of cardiac death, non-fatal myocardial infarction, and urgent revascularization. Secondary endpoint included rehospitalization owing to ACS, heart failure, stroke, and other thrombotic events. The Seattle Angina Questionnaire (SAQ) was used to assess quality of life (QOL). Primary and secondary endpoints were followed up for 36 months, and the SAQ was followed up for 12 months. The Cox proportional hazards regression model was used to analyze the independent effect of DLT on primary and secondary endpoints. Propensity score matching (PSM) analyses were performed to mitigate bias. Survival estimation was performed using Kaplan–Meier survival curves and log-rank tests in the PSM cohort, and landmark analyses were used for further evaluation of primary and secondary endpoints. Subgroup analyses and interactions confirmed the robustness of the findings. Linear mixed effects models were used to assess the QOL.

**Results:**

Overall, 936 patients were enrolled in this cohort study, of whom 875 completed follow-up. The primary and secondary endpoints had no significantly difference between the DLT and CM groups after Cox proportional hazards models. Kaplan–Meier survival curves and log-rank tests performed in the PSM cohort also found no significant differences between the two groups on primary and secondary endpoints. However, landmark analysis showed significant benefit in the primary endpoint for the DLT group after 200 days (hazard ratio [HR] 0.46, 95% confidence interval [CI] 0.22–0.93, *P *= 0.03). Landmark analysis also showed a significant benefit in the secondary endpoint in the DLT group within 200 days (HR 0.33, 95% CI 0.15–0.73, *P *= 0.006). Moreover, DLT improves the SAQ summary score, and scores in the physical limitation, treatment satisfaction, and disease perception domains for patients with ACS undergoing PCI.

**Conclusions:**

DLT combined with conventional treatment reduced the risk of the primary endpoint after 200 days and the secondary endpoint within 200 days during the 3-year follow-up. Additionally, DLT can improve the QOL without adverse effects.

## 1 Introduction

Acute coronary syndrome (ACS) poses a significant risk to public health and is the primary contributor to increased in cardiovascular mortality (1). An estimated 7 million individuals globally are diagnosed with ACS each year (2). Percutaneous coronary intervention (PCI) has become a the routine treatment for ACS revascularization, reducing the mortality of patients with ACS. However, PCI itself can cause endothelial damage and platelet activation, and the use of conventional anticoagulation and antiplatelet drugs post-PCI carries a risk of thrombosis and bleeding, which may increase the likelihood of cardiovascular events (3, 4). Statistically, the incidence of major cardiovascular events in patients post-PCI is as high as 20 percent over a 3-year period, despite adherence to secondary prevention medication following the recommendations of the guidelines (5). Therefore, further reduction of the risk of cardiovascular events after PCI further has become a critical issue that needs to be urgently addressed.

Chinese medicine has gained increasing recognition worldwide owing to its multi-target and multi-level functional characteristics. For many years, China authorized the use of the patented Chinese medication Danlou tablet (DLT) for thetreatment of coronary heart disease (CHD). DLT is composed of 10 kinds of Chinese herbs: *Trichosantheskirilowii, Allium macrostemon, Pueraria montana var. lobata, Conioselinumanthriscoides, Salvia miltiorrhiza, Paeonia anomala subsp. Veitchii, Alisma plantago-aquatica subsp. Orientale, Astragalus mongholicus, Drynariaroosii,* and *Curcuma aromatica* (6). Previous research has demonstrated that DLT protects the heart against remodeling and dysfunction during myocardial ischemia/reperfusion injury in rats by stimulating the AKT/FoxO3a pathway (7). A clinical study has found that DLT reduces the risk of cardiovascular events in patients with non-ST-segment elevation acute coronary syndromes (NSTE-ACS) undergoing PCI (8). According to a meta-analysis of 17 randomized controlled trials, combining DLT with conventional medicine (CM) can reduce the frequency and duration of angina pectoris, demonstrating positive efficacy and safety (9). However, whether DLT combined with CM can reduce the risk of cardiovascular events in ACS patients undergoing PCI remains to be fully investigated. Therefore, we conducted a multicenter prospective cohort study to investigate the effect of DLT in combination with CM on cardiovascular endpoint in patients with ACS undergoing PCI.

## 2 Methods

### 2.1 Ethics statement

This clinical trial constitutes a subset of the CPM trial and has been registered with the Chinese Clinical Trial Registry (http://www.chictr.org.cn) under registration number ChiCTR-OOC-14005552. The design and protocol of this trial were approved by the Ethics Committee of Xiyuan Hospital, China Academy of Chinese Medical Sciences (No.2011XL017-2), and the respective Ethics Committees of all participating centers. Informed consent was obtained from all enrolled patients. This study was conducted in accordance with the Declaration of Helsinki and adhered to the Strengthening the Reporting of Observational Studies in Epidemiology (STROBE) guidelines.

### 2.2 Study design and participants

This prospective cohort study was conducted at 40 Chinese medical facilities. It enrolled patients with ACS who underwent PCI for the first time between February 2012 and December 2015. The inclusion criteria were: (i) patients with ACS undergoing PCI for the first time, (ii) patients aged of 18–75 years, and (iii) patients diagnosed with phlegm-stasis obstructive syndrome according to traditional Chinese medicine differentiation. Detailed criteria have been described in our previous research (10).

### 2.3 Data collection

Patients in this cohort were divided into the DLT and the CM groups based on their use of DLT prior to enrollment. The DLT group received DLT (1.5 g, three times daily for 12 months) alongside CM treatment. Baseline characteristics of patients in both groups were recorded, encompassing gender, age, family history, heart rate, blood pressure, comorbidities, concomitant medications, number of diseased vessels, and subtype of ACS.

### 2.4 Outcome measures

The primary endpoint was a composite outcome that included cardiac death, non –fatal myocardial infarction, and urgent revascularization. The secondary endpoint encompassed rehospitalization owing to ACS, heart failure, stroke, and other thrombotic events. Both the primary and secondary endpoints were treated as time-to-event data. An outcome was defined as the first occurrence of any of these events during the 3-year follow-up period, as detailed in Supplementary Material and our previous publication (10). Quality of life (QOL) was assessed using the Seattle Angina Questionnaire (SAQ), which consists of of five domains: physical limitation (PL), angina stability (AS), angina frequency (AF), treatment satisfaction (TS), and disease perception (DP). SAQ scores were collected at baseline and 1, 3, 6, and 12 months of follow-up, with higher scores indicating better QOL. Any discomfort or abnormal laboratory results deemed clinically significant after enrollment, in addition to the primary and secondary outcomes, were designated as adverse events (AEs).

### 2.5 Statistical analysis

Appropriate statistical methods for cohort studies were employed to analyze the collected data. Continuous variables are expressed as means ± standard deviation (SD) or as medians and interquartile range, depending on the data distribution determined by normality testing. Frequency and percentage were used to represent categorical variables. Mann-Whitney *U* tests, chi-square tests, and *t*-tests were used to compare the two groups’ baselines.

Cox regression models were used to evaluate the relationship between the outcomes and the exposures. This study presents four Cox regression models in accordance with the STROBE statement. Model 1 includes no adjustment for confounders, while Model 2 adjusts for gender and age. Model 3 further adjusts for confounders identified in model 2 based on changes in estimates (CIE), and Model 4 adjusts for all confounders.

To mitigate confounders resulting from non-randomization in this observational study, we employed propensity score matching (PSM) to balance all baseline characteristics between the two groups in a 1:1 ratio, with nearest neighbour matching selected as the matching method. All baseline characteristics of the two groups listed in Table 1 were used to create the propensity score. The match tolerance was set to 0.05. Subsequently, univariate regression and covariate adjustment using propensity score (CAPS) were performed in the matched cohort. Univariate regression did not involve adjustment for confounders, whereas CAPS included propensity score as a covariate adjustment.

**Table 1:** Baseline characteristics of participants with ACS undergoing PCI and received DLT or not.

Characteristic	Before propensity score matching			After propensity score matching		
	DLT group (*n* = 443)	CM group (*n* = 432)	*P* value	DLT group (*n* = 322)	CM group (*n* = 322)	*P* value
Gender						
Male (*N*/%)	328 (74.04)	308 (71.30)	0.362	234 (72.67)	229 (71.12)	0.661
Female (*N*/%)	115 (25.96)	124 (28.70)		88 (27.33)	93 (28.88)	
Age (y)	60.00 (52.50–67.00)	60.00 (53.00–67.00)	0.799	60.00 (53.25–67.00)	59.00 (53.00–66.75)	0.741
Family history (*N*/%)	143 (32.28)	131 (30.32)	0.707	101 (31.37)	96 (29.81)	0.771
Currently smoking (*N*/%)	301 (67.95)	301 (69.68)	0.581	219 (68.01)	225 (69.88)	0.609
Currently drinking (*N*/%)	119 (26.86)	111 (25.69)	0.695	74 (22.98)	74 (22.98)	1.000
Heart rate (bpm)	72.00 (64.00–78.00)	72.00 (68.00–80.00)	0.003	72.00 (64.25–78.00)	72.00 (68.00–80.00)	0.049
Sinus rhythm (*N*/%)	433 (97.74)	418 (96.76)	0.373	312 (96.89)	311 (96.58)	0.824
Body mass index (kg/m^2^)	24.77 (23.14–27.04)	25.21 (23.51–27.72)	0.010	24.95 (23.14–27.34)	25.12 (23.53–27.78)	0.075
Blood pressure (mmHg)						
SBP	130.00 (120.00–140.00)	130.00 (120.00–140.00)	0.098	130.00 (120.00–140.00)	130.00 (120.00–140.00)	0.221
DBP	80.00 (70.00–84.00)	80.00 (70.00–90.00)	0.056	80.00 (70.00–85.00)	80.00 (70.25–90.00)	0.186
Comorbidities, (*N*/%)						
Hypertension	265 (59.82)	282 (65.28)	0.095	204 (63.35)	212 (65.84)	0.510
Type 2 diabetes	197 (44.47)	158 (36.57)	0.017	141 (43.79)	115 (35.71)	0.036
Hyperlipidemia	257 (58.01)	289 (66.90)	0.007	204 (63.35)	218 (67.70)	0.246
Stroke	14 (3.16)	124 (28.70)	<0.001	14 (4.35)	15 (4.66)	0.849
Medications (*N*/%)						
Beta-blockers	137 (30.93)	114 (26.39)	0.138	98 (30.43)	83 (25.78)	0.189
ACEIs	178 (40.18)	131 (30.32)	0.002	114 (35.40)	93 (28.88)	0.076
ARBs	107 (24.15)	131 (30.32)	0.040	88 (27.33)	99 (30.75)	0.340
CCBs	90 (20.32)	68 (15.74)	0.079	69 (21.43)	51 (15.84)	0.069
Statins	279 (62.98)	255 (59.03)	0.231	196 (60.87)	192 (59.63)	0.747
ACS subtype, (*N*/%)						
NSTE-ACS	335 (75.62)	343 (79.40)	0.181	247 (76.71)	260 (80.75)	0.211
STE-ACS	108 (24.38)	89 (20.60)		75 (23.29)	62 (19.25)	
Number of diseased vessels, (*N*/%)						
One	192 (43.34)	192 (44.44)	0.699	138 (42.86)	144 (44.72)	0.628
Two	230 (51.92)	215 (49.77)		170 (52.80)	160 (49.69)	
Three	21 (4.74)	25 (5.79)		14 (4.35)	18 (5.59)	
Diseased coronary artery, (*N*/%)						
LAD	251 (56.66)	235 (54.40)	0.501	183 (56.83)	172 (53.42)	0.383
LCX	258 (58.24)	248 (57.41)	0.803	188 (58.39)	181 (56.21)	0.577
RCA	156 (35.21)	156 (36.11)	0.782	113 (35.09)	124 (38.51)	0.369
LM	51 (11.51)	58 (13.43)	0.391	37 (11.49)	42 (13.04)	0.548

SBP, systolic blood pressure; DBP, diastolic blood pressure; ACEI, angiotensin-converting enzyme inhibitors; ARB, angiotensin II receptor blockers; CCBs, calcium channel blockers; ACS, acute coronary syndromes; NSTE, non-ST elevation; STE, ST elevation; LAD, left anterior descending artery; LCX, left circumflex coronary artery; RCA, right coronary artery; LM, left main coronary artery; DLT, Danlou tablets; CM, conventional medicine.

To account for baseline differences between patients with DLT and CM, propensity score–based matching was used. Propensity scores were calculated in each database independently, based on available demographic characteristics, as well as the comorbidities, medication, type of ACS, number of diseased vessels, and diseased coronary artery of each database. *P*-value refers to a comparison between subjects in the DLT group and the CM group.

This study analyzed the time from enrollment to the occurrence of the first event related to both the primary and secondary endpoints. Kaplan–Meier (K–M) survival curves were used to estimate survival probabilities, complemented by the log–rank test. Additionally, a landmark analysis was conducted for both primary and secondary endpoints, using a time cut-off of 200 days.

All statistical analyses were performed using the R 4.0.5 with data anonymized.

## 3 Results

### 3.1 Cohort characteristics

**Figure 1:**
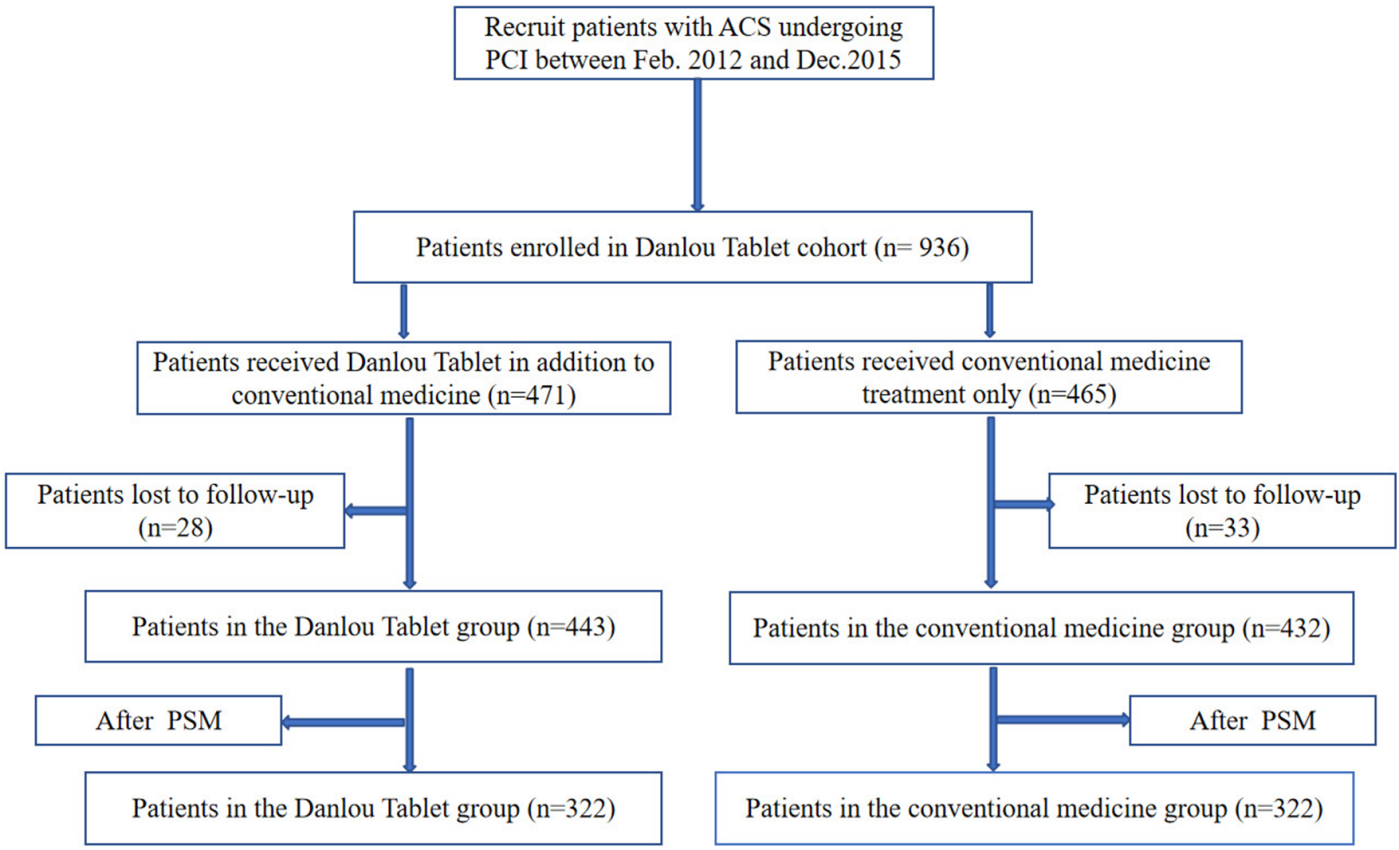
Patient enrolment and follow-up.

A total of 936 patients diagnosed with ACS and undergoing PCI were identified as meeting the inclusion criteria for this study. Among these patients, 471 were in the DLT group, and 465 were in the CM group. The follow-up rate was 93.48%, with 61 patients lost to follow-up. Finally, 443 patients in the DLT group and 432 patients in the CM group were included in the statistical analysis. The cohort flow diagram is presented in Figure 1. The baseline clinical characteristics of the study population before and after PSM are summarized in Table 1. Differences were observed across certain covariates before PSM. However, after PSM, there were no significant differences in baseline clinical characteristics except for heart rate and the presence of type 2 diabetes mellitus.

### 3.2 Primary endpoint

**Figure 2:**
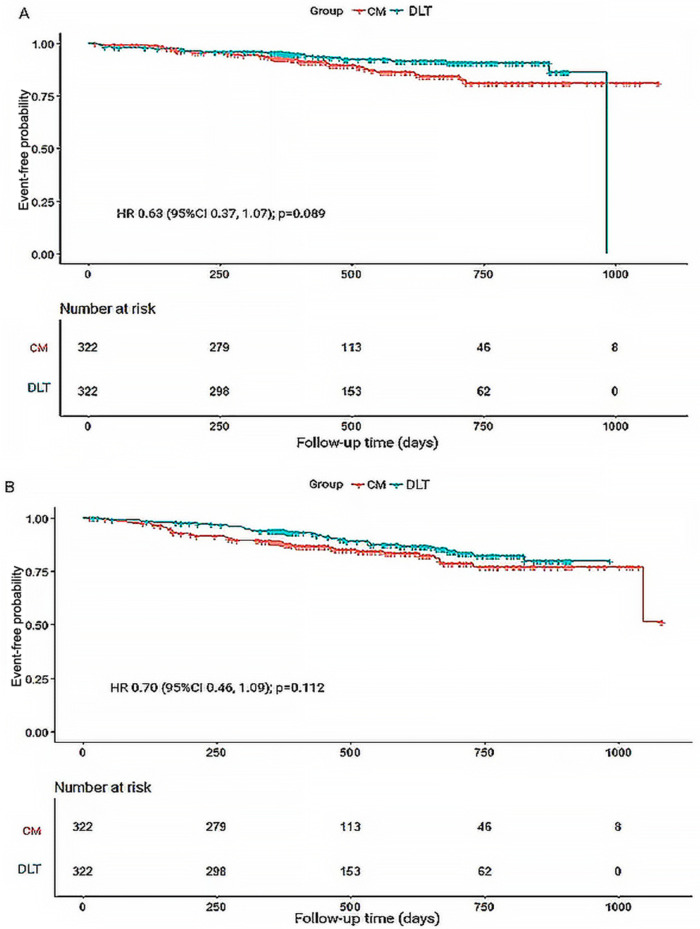
Kaplan-Meier survival curves for primary and secondary endpoints during 3-year follow-up. **(A)** Kaplan-Meier survival curves for primary endpoint-free probability. **(B)** Kaplan-Meier survival curves for secondary endpoint-free probability. HR, hazard ratio.

**Figure 3:**
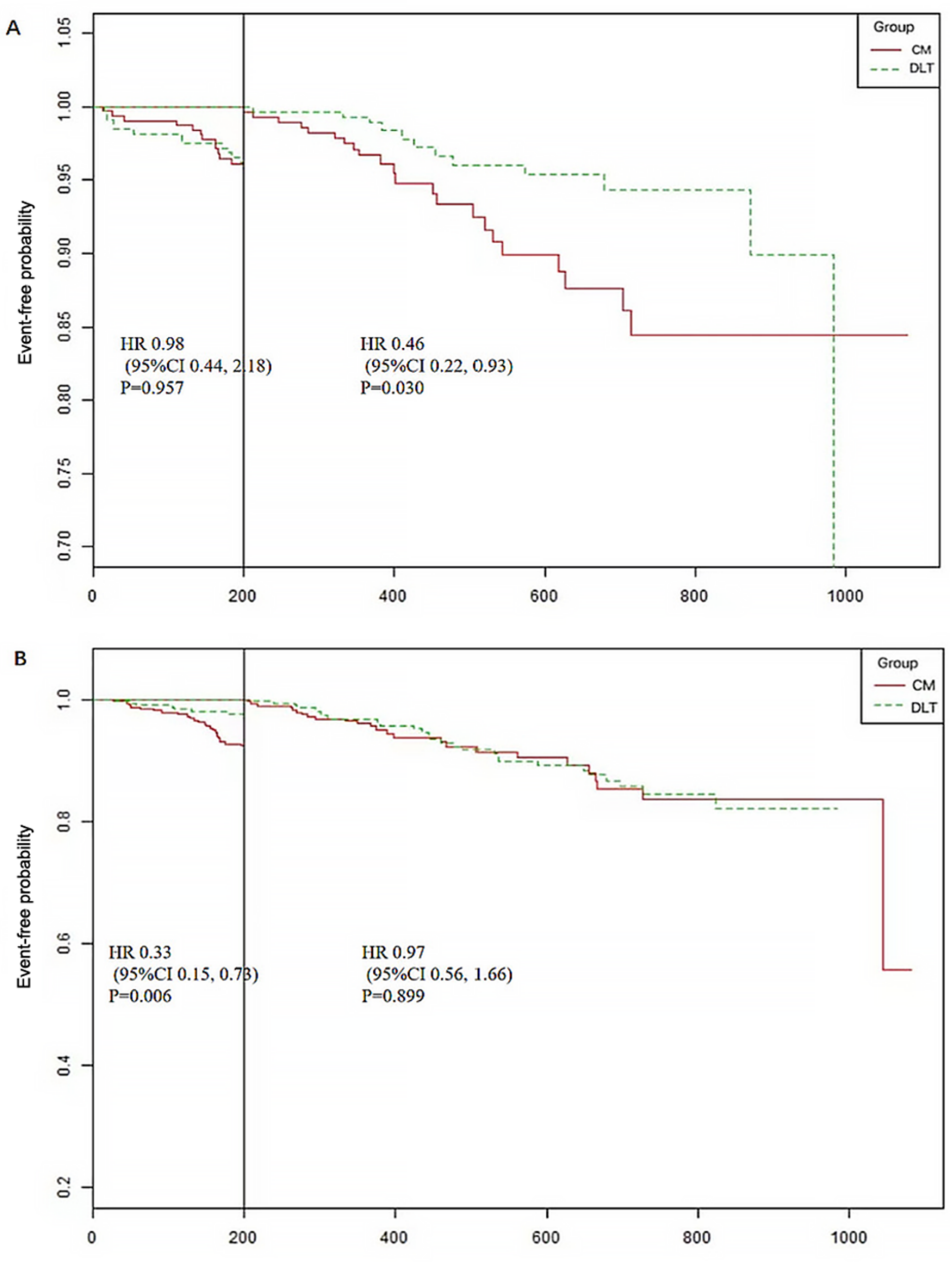
Landmark analysis of the primary and secondary endpoints (time cut point 200 days). **(A)** The primary endpoint includes cardiac death, nonfatal myocardial infarction, and urgent revascularization. **(B)** Secondary endpoint includes rehospitalization for ACS, heart failure, stroke and other thrombotic events.

A total of 44 patients (10.19%) in the CM group and 35 (7.90%) in the DLT group reached the primary endpoint during the 3-year follow-up. The risk of the primary endpoint did not differ significantly between DLT and CM groups after adjusting for confounders using Cox proportional hazards models (hazard ratio [HR] 0.68, 95% confidence interval [CI] 0.42–1.12, *P* = 0.134), in accordance with the analysis after PSM (HR 0.63, 95% CI 0.37–1.07, *P* = 0.089) (Table 2). However, landmark analysis showed meaningful benefit for the primary endpoint in the DLT group after 200 days (HR 0.46, 95% CI 0.22–0.93, *P *= 0.030) (Table 3). The survival curves for the 3-year primary endpoint after PSM are presented in Figure 2A. Landmark analysis of the primary endpoint in the PSM cohort is depicted in Figure 3A.

**Table 2:** Comparison of primary and secondary endpoints between patients in the DLT and CM groups before and after propensity score matching.

	Before propensity score matching			
	Primary endpoint	*P* value	Secondary endpoint	*P* value
DLT group (*n* = 443,%)	35 (7.90)		57 (12.87)	
CM group (*n* = 432,%)	44 (10.19)		63 (14.58)	
Model 1, HR (95% CI)	0.69 (0.44, 1.08)	0.105	0.79 (0.55, 1.13)	0.193
Model 2, HR (95% CI)	0.67 (0.43, 1.05)	0.084	0.78 (0.55, 1.13)	0.189
Model 3, HR (95% CI)	0.67 (0.41, 1.07)	0.095	0.77 (0.52, 1.14)	0.197
Model 4, HR (95% CI)	0.68 (0.42, 1.12)	0.134	0.81 (0.54, 1.23)	0.326
** **	After propensity score matching			
	Primary endpoint	*P* value	Secondary endpoint	*P* value
DLT group (*n* = 322,%)	24 (7.45)		37 (11.49)	
CM group (*n* = 322,%)	34 (10.56)		49 (15.22)	
Univariate regression, HR (95% CI)	0.63 (0.37, 1.07)	0.087	0.66 (0.43, 1.02)	0.062
CAPS, HR (95% CI)	0.63 (0.37, 1.07)	0.089	0.70 (0.46, 1.09)	0.112

DLT, Danlou tablet; CM, conventional medicine; CAPS, covariate adjustment using propensity score; HR, hazard ratio; CI, confidence interval.

There is no adjustment for any confounders in model 1. Gender and age are adjusted in model 2. Variables in model 3 are adjusted based on the change in estimate method. The primary endpoint model adjustment variables include gender, age, hyperlipidemia and stroke. The secondary endpoint model adjusted variables were gender, age, BMI, ACEI, CCB and stroke. All confounders were adjusted for in model 4.

There is no adjustment for any confounders in the univariate regression. Propensity score adjusted as a covariate in the CAPS.

*P*-value refers to comparison between subjects in the DLT group and the CM group.

**Table 3:** Risk of primary and secondary endpoints after landmark analysis.

Events	HR (95% CI)	*P* value
Primary endpoint (<200 days)	0.98 (0.44, 2.18)	0.957
Primary endpoint (≥200 days)	0.46 (0.22, 0.93)	0.030
Secondary endpoint (<200 days)	0.33 (0.15, 0.73)	0.006
Secondary endpoint (≥200 days)	0.97 (0.56, 1.66)	0.899

HR, hazard ratio; CI, confidence interval.

*P*-value refers to a comparison between subjects in the DLT group and the CM group after landmark analysis.

### 3.3 Secondary endpoint

The incidence of secondary endpoint was 14.58% (*n* = 63) in the CM group and 12.87% (*n* = 57) in the DLT group. The risk of the secondary endpoint did not differ significantly between DLT and CM groups after Cox proportional hazard models (HR 0.81, 95% CI 0.54–1.23, *P* = 0.326), in conformity with the analysis after PSM (HR 0.70, 95% CI 0.46–1.09, *P* = 0.112) (Table 2). However, landmark analysis showed a significant difference in the secondary endpoint in the DLT group within 200 days (HR 0.33, 95%CI 0.15–0.73, *P *= 0.006) (Table 3). The survival curves for the 3-year secondary endpoint after PSM are presented in Figure 2B, and the landmark analysis of the secondary endpoint in the PSM cohort is presented in Figure 3B.

### 3.4 QOL

SAQ and its subdomains were used to evaluate the QOL in the DLT and CM groups (Supplementary Table S1; Supplementary Figure S1). Linear mixed-effect models were applied to account for interactions between time and group. The findings showed that DLT significantly improved the QOL in the summary score (*β*13.19, 95% CI 4.03–22.35, *P* = 0.005), PL (*β*2.94, 95% CI 0.75–5.13, *P* = 0.009), TS (*β*2.27, 95% CI 0.16–4.38, *P* = 0.035), and DP domains (*β*2.93, 95% CI 0.51–5.36, *P* = 0.018) for patients with ACS undergoing PCI (Supplementary Table S2). No significant interaction was observed between the time and group variables in the SAQ total score and its PL, TS, and DP subdomains (*P* > 0.05) (Supplementary Table S2).

### 3.5 Primary and secondary endpoints in selected subgroups

**Figure 4:**
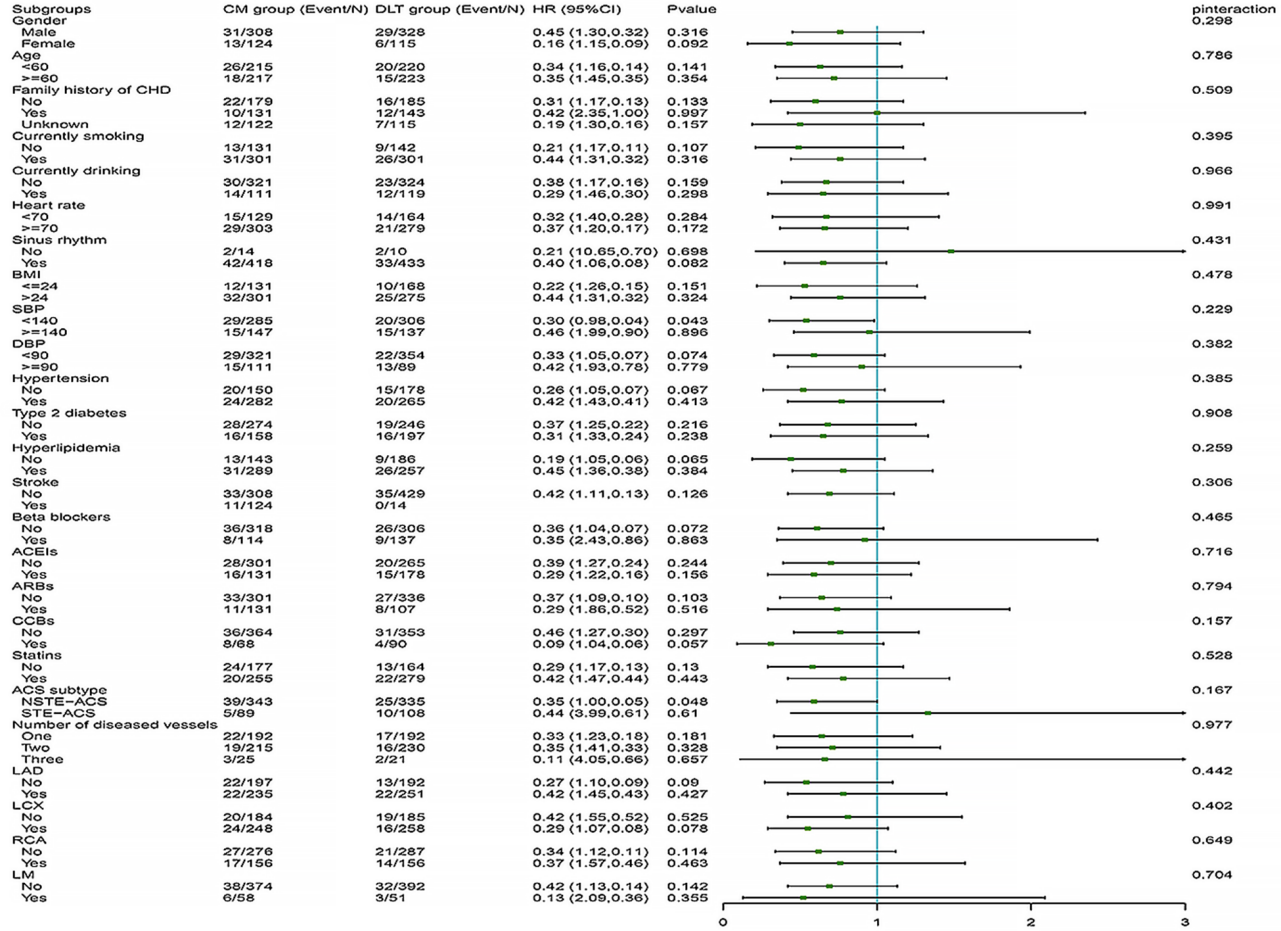
Primary endpoint in selected subgroups. The HRs for the primary efficacy endpoint in both groups are shown according to prespecified and post-hoc subgroups. HR, hazard ratio; DLT, Danlou tablet; CM, conventional medicine.

**Figure 5:**
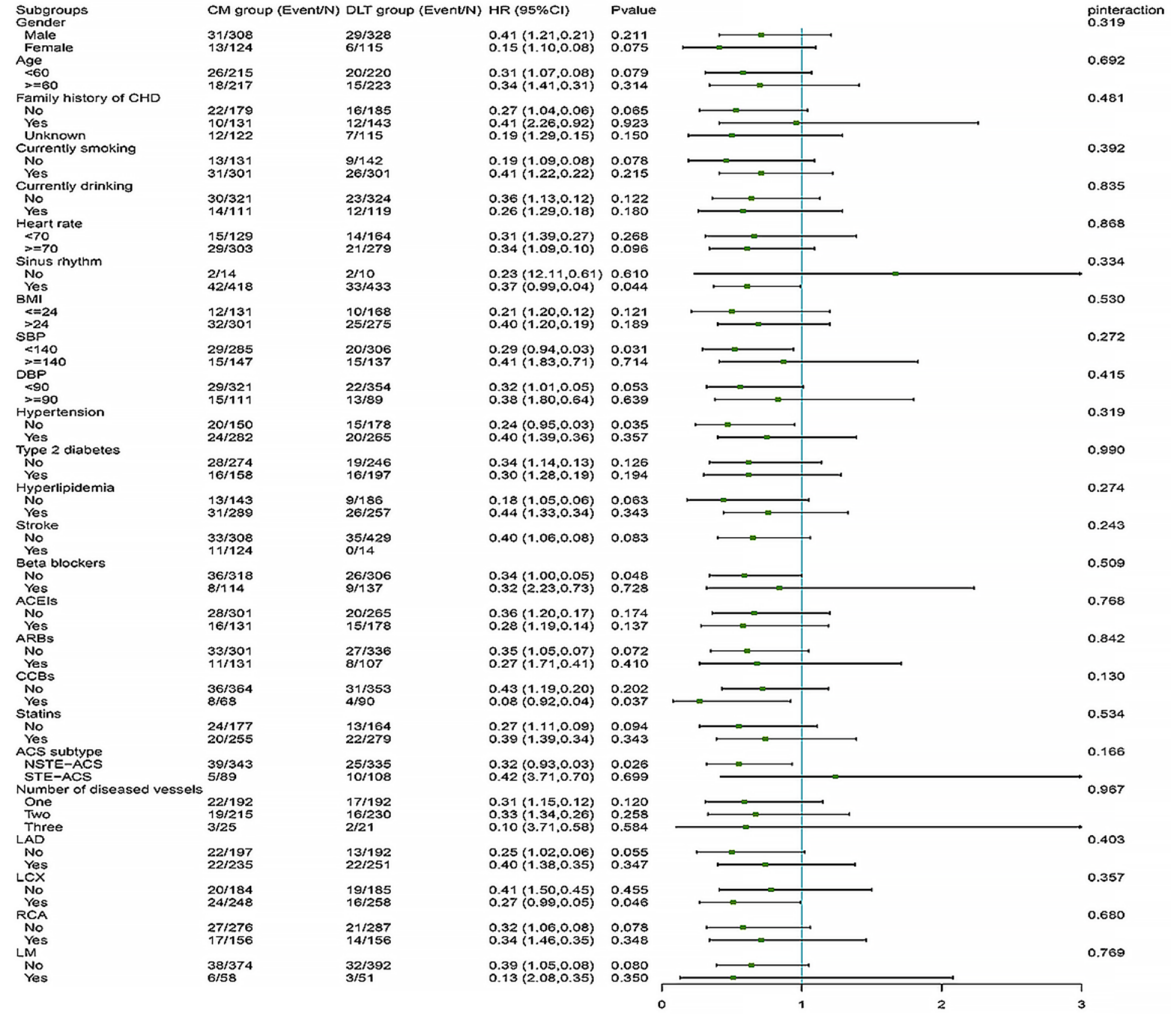
Secondary endpoint in selected subgroups. The HRs for the secondary efficacy endpoint in both groups are shown according to prespecified and post-hoc subgroups. HR, hazard ratio; DLT, Danlou tablet; CM, conventional medicine.

The difference in efficacy between the DLT and CM groups for the primary and secondary endpoints was not statistically significant in most of the pre-specified subgroups. However, in subgroups with systolic blood pressure reduction and the NSTE-ACS subtype, the DLT group demonstrated better efficacy than the CM group for primary endpoint (Figure 4). Meanwhile, in subgroups with sinus rhythm, systolic blood pressure reduction, nonhypertensive, beta-blockers, CCBs use, NSTE-ACS subtype, and left circumflex branch disease, the DLT group demonstrated better efficacy than the CM group for secondary endpoint (Figure 5). Post-hoc analysis showed no significant interaction between the treatment effect and any of the subgroups.

### 3.6 Safety

During the follow-up period, 34 (7.67%) adverse events occurred in the DLT and 32 (7.41%) in the CM groups (Supplementary Table S3). In addition, no significant differences were observed in the incidence of adverse events in the two groups (*P *= 0.806). Diarrhea was the most frequent adverse event, but no major adverse events were reported. In addition, there was no patients withdrew from the trial owing to adverse events.

## 4 Discussion

To the best of our knowledge, this study represents the largest published investigation to date on the cardiovascular event efficacy of DLT in patients with ACS undergoing PCI. A relatively large sample size is essential in drawing reliable conclusions. Our findings indicate that while DLT did not reduce the risk of primary and secondary endpoints during 3-year follow-up compared to conventional treatment, it did reduce the risk of the secondary endpoint within 200 days and the primary endpoint after 200 days. In addition, DLT increased the total SAQ score and its PL, TS, and DP subdomain, thereby improving the QOL in ACS patients treated with PCI.

Previous studies have found that DLT reduces the risk of non-fatal myocardial infarction in NSTE-ACS patients treated with PCI compared to placebo (8). Non-fatal myocardial infarction was a component of the primary endpoint observed in this study. Furthermore, DLT was found to reduce the risk of the primary endpoint after 200 days in landmark analysis, which is consistent with previous findings. Additionally, Wang et al. found that DLT can reduce the duration and frequency of chest tightness and pain, and improve angina score (11). A meta-analysis that included 17 randomized controlled trials showed that DLT combined with conventional Western medical treatment reduced the duration and frequency of angina episodes and improved the degree of angina (9). In this study, DLT can significantly increased the total SAQ score and its PL, TS, and DP subdomain scores, indicating that DLT improved the QOL in patients with ACS undergoing PCI, in accordance with previous research.

Basic studies have found that DLT can not only prevent myocardial infarction by promoting eNOS-dependent endothelial protection and angiogenesis (12) but also attenuates myocardial ischemia/reperfusion injury by activating the AKT/FoxO3a (7) and PPARγ (13) pathways. This may be the potential mechanism by which DLT reduces the risk of endpoints and improves SAQ scores. In addition, the beneficial effects of DLT may also be attributed to the synergistic action of the drugs in the formula. The Gualou-Xiebai herb pair inhibits ferroptosis-mediated endothelial cell injury by activating the Nrf2 signaling pathway and further alleviates atherosclerosis pathological damage (14). It has been demonstrated that puerarin and its derivatives, including puerarin V, lessen myocardial damage and apoptosis, hence preventing and regulating myocardial infarction in stages. The mechanisms involved include activation of the PI3K/Akt/GSK-3β pathway, suppression of inflammation, and stimulation of cardiac PPARγ expression (15). Network pharmacology and molecular docking studies have shownthat the combination of Astragalus membranaceus and Ligusticum chuanxiong can potentially inhibit inflammatory responses, reduce lipid accumulation, delay the progression of atherosclerosis, and consequently, enhance circulation to alleviate ischemia (16). Danshensu IIA reprograms macrophage phenotypes post-myocardial infarction by intervening in the PGK1-PDHK1 pathway, thereby inhibiting inflammatory responses (17). The combination of Tanshinone IIA and Astragaloside IV attenuates myocardial ischemia-reperfusion injury by inhibiting the STING pathway (18). Chishao terpene glycoside is a potential candidate drug for preventing and treating myocardial ischemia, as it improves cardiac energy metabolism and inhibits myocardial cell apoptosis by activating the PI3K/AKT/mTOR signaling pathway (19). Alisol B 23-acetate improves myocardial function by inhibiting inflammation and reactive oxygen species production through the TLR4/NOX2 pathway (20). These pharmacological effects collectively contribute to the reduction of cardiovascular event risk and the improvement in QOL by DLT.

The incidence of the primary endpoint in our study is differs from that reported in previous studies. This variance may be attributed to differences in the study populations, observation periods, and definitions of endpoints. Sanjit et al. (21) included patients with ST-elevation myocardial infarction and followed up for 12 months to observe cardiovascular events. The lower incidence of cardiovascular endpoints in their study compared to our current research may be owing to the shorter follow-up period, and the cardiovascular events included cardiovascular death, recurrent MI, and unplanned ischemia-driven target vessel revascularization. In another study conducted by Gan et al. (22), major cardiovascular events included acute myocardial infarction, recurrent chest pain, heart failure, stroke, revascularization, and cardiac death, which do not entirely align with our definition of cardiovascular events. These inconsistent definitions of endpoints may contribute to disparities in cardiovascular event risk observed between their study and our current research.

This study was observational research, which inevitably leads to a significant imbalance between the two groups at baseline. To address this, PSM was employed to match baseline characteristics and minimize differences between the two groups to mitigate the interference of baseline confounders. After PSM, comparability was achieved between the baseline characteristics of the two groups except for heart rate and concomitant diabetes. Additionally, regression equations were utilized within the matched cohort to further control for confounders. K–M survival curves and log-rank tests were employed to assess the impact of grouping on outcomes, considering survival time, as is commonly utilized in survival analysis. While the log-rank test yielded non-significant results, significant differences in the slope of the K–M survival curves were observed at approximately 200 days, suggesting differential intervention effects on outcomes before and after this period. Therefore, a landmark analysis was conducted using 200 days as a cutoff point to dynamically monitor the efficacy of DLT at different time intervals, providing insights for clinical treatment strategies. Furthermore, SAQ scores were measured at baseline, 1, 3, 6, and 12 months, constituting repeated measures data. Since individual observations of repeated measures data are not entirely independent, analyzing them using statistical inference methods for independent data may increase the probability of type I errors. Hence, a linear mixed-effects model, a commonly used statistical approach for repeated measures data, was employed to compare SAQ scores between the two groups; this approach was adopted to ensure the robustness and reliability of our findings.

The incidence of adverse events did not significantly differ between the DLT and CM groups, and no serious adverse events were reported. This indicates that the safety profile of DLT is acceptable, which is in line with findings from previous research (23).

Even so, this study also has some limitations that warrant discussion. Firstly, cohort studies inherently have more confounders and biases compared to randomized controlled trials because of the difficulties of randomization and blinding. To address this, we employed PSM to balance the baseline characteristics between the two groups, ensuring the validity of the results. Additionally, the statistical analysis of the data was blinded. Secondly, despite no significant differences in primary and secondary endpoints between the two groups over the 3-year follow-up, landmark analysis showed that DLT was associated with a reduced risk of the primary endpoint after 200 days and the secondary endpoint within 200 days. This discrepancy may be related to the nature of the endpoints themselves. Specifically, the secondary endpoint defined in this study included more immediate and potentially reversible events, such as rehospitalization owing to ACS, heart failure, and other thrombotic events. The effect of DLT on these endpoints may be observed more quickly over a shorter period. In contrast, the primary endpoint consists of more serious and definitive events, such as cardiac death, non-fatal myocardial infarction, and urgent revascularization, which are usually influenced by long-term treatment rather than immediate intervention. The beneficial effects of DLT on these more serious outcomes became apparent only after long-term treatment, which is consistent with the observed benefits after 200 days. Furthermore, the effect of DLT at different times on different endpoint events informs clinical decision-making and guideline development. Thirdly, the study involved multiple medical centers, and there may be subjective bias in the scoring of the SAQ. Therefore, c onsistency training should be enhanced in future studies to obtain more reliable conclusions. Then, one limitation of this study is the exclusion of residual angina at baseline from the baseline characteristics. To address this, the total SAQ (Seattle Angina Questionnaire) score and the scores for each domain were recorded at baseline and at corresponding follow-up time points to assess patients’ quality of life. The SAQ consists of five domains, including angina frequency and angina stability. Changes in angina frequency and stability scores before and after DLT treatment may serve as a reference for evaluating the effectiveness of DLT in treating angina. In addition, the lack of precise information on the duration of DLT use prior to enrollment is a limitation of this study. To better observe the long-term effectiveness and safety of DLT, patients in the DLT group underwent a 12-month course of DLT treatment following enrollment. Finally, all patients enrolled in the current study were of Chinese ethnicity. Further study is needed to verify whether our findings are applicable to patients from different racial backgrounds.

## 5 Conclusion

Among patients with ACS undergoing PCI in routine clinical practice, DLT combined with CM was associated with a lower risk of the primary endpoint after 200 days and the secondary endpoint within 200 days during a 3-year follow-up. In addition, DLT improves QOL in these patients. Given the inherent limitations of cohort studies, it is recommended that future studies expand the sample size and employ randomized controlled trials to further confirm the clinical efficacy of DLT.

## Data Availability

The original contributions presented in the study are included in the article/Supplementary Material, further inquiries can be directed to the corresponding authors.
