# Supplementary material for: Efficacy of Danlou tablets in patients with acute coronary syndrome undergoing percutaneous coronary intervention: a multicenter prospective cohort study
Source: Front Cardiovasc Med. 2024 Sep 24;11:1420194. doi: 10.3389/fcvm.2024.1420194 (PMC11458459; doi:10.3389/fcvm.2024.1420194)
Supplement: Supplementary file 1 [file Datasheet1.docx]

Supplementary Material

Table S1 Comparison of Health Status Between DLT group and CM group

|  | DLT Group | | CM Group | | *P* value |
| --- | --- | --- | --- | --- | --- |
|  | Mean | SD | Mean | SD |  |
| Baseline |  |  |  |  |  |
| Summary score | 275.61 | 94.88 | 260.55 | 87.67 | 0.012 |
| Physical limitation | 51.55 | 21.82 | 49.02 | 21.03 | 0.072 |
| Angina stability | 47.13 | 41.73 | 40.86 | 40.17 | 0.019 |
| Angina frequency | 62.14 | 27.95 | 60.47 | 26.76 | 0.350 |
| Treatment satisfaction | 67.57 | 21.04 | 65.72 | 19.97 | 0.169 |
| Disease perception | 47.22 | 22.62 | 44.48 | 19.81 | 0.049 |
| 1-month |  |  |  |  |  |
| Summary score | 370.30 | 70.18 | 353.57 | 75.51 | 0.001 |
| Physical limitation | 64.18 | 16.77 | 60.29 | 17.70 | 0.001 |
| Angina stability | 86.62 | 25.19 | 84.73 | 26.43 | 0.262 |
| Angina frequency | 84.23 | 19.09 | 80.95 | 21.42 | 0.014 |
| Treatment satisfaction | 75.32 | 15.62 | 71.90 | 17.19 | 0.002 |
| Disease perception | 59.94 | 19.90 | 55.70 | 18.99 | 0.001 |
| 3-month |  |  |  |  |  |
| Summary score | 369.98 | 70.14 | 357.07 | 74.74 | 0.008 |
| Physical limitation | 64.41 | 16.79 | 61.24 | 17.54 | 0.006 |
| Angina stability | 86.17 | 25.61 | 84.87 | 25.65 | 0.447 |
| Angina frequency | 84.04 | 19.61 | 81.75 | 21.26 | 0.093 |
| Treatment satisfaction | 75.24 | 15.71 | 72.78 | 16.89 | 0.024 |
| Disease perception | 60.11 | 19.45 | 56.43 | 19.43 | 0.005 |
| 6-month |  |  |  |  |  |
| Summary score | 372.28 | 68.24 | 357.82 | 74.67 | 0.003 |
| Physical limitation | 64.54 | 16.80 | 61.15 | 17.51 | 0.004 |
| Angina stability | 87.22 | 24.63 | 85.34 | 25.26 | 0.268 |
| Angina frequency | 84.57 | 18.93 | 82.03 | 21.39 | 0.065 |
| Treatment satisfaction | 75.80 | 15.38 | 72.66 | 17.11 | 0.005 |
| Disease perception | 60.15 | 19.43 | 56.64 | 19.24 | 0.008 |
| 12-month |  |  |  |  |  |
| Summary score | 369.43 | 68.86 | 352.18 | 77.31 | 0.002 |
| Physical limitation | 63.63 | 16.87 | 59.99 | 17.65 | 0.005 |
| Angina stability | 87.17 | 24.73 | 84.29 | 26.71 | 0.132 |
| Angina frequency | 84.00 | 19.24 | 81.04 | 22.01 | 0.055 |
| Treatment satisfaction | 75.73 | 15.71 | 71.64 | 17.58 | 0.001 |
| Disease perception | 58.90 | 19.32 | 55.21 | 19.27 | 0.010 |

Abbreviation: SD, standard deviation. P-value refers to comparison between subjects with DLT group and CM group.


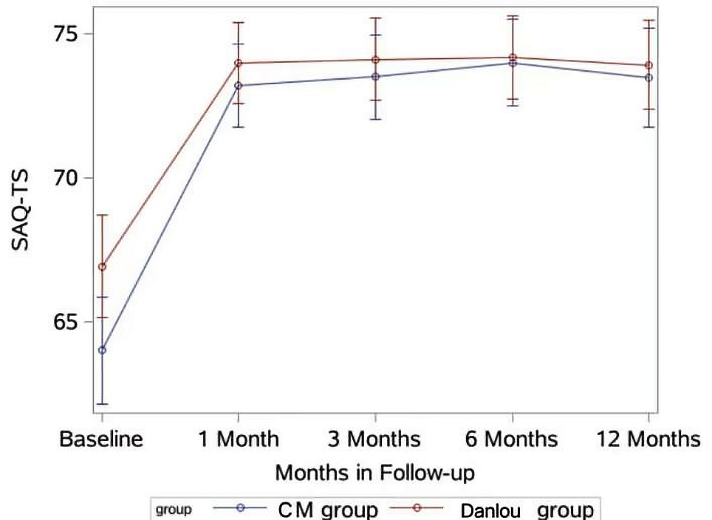

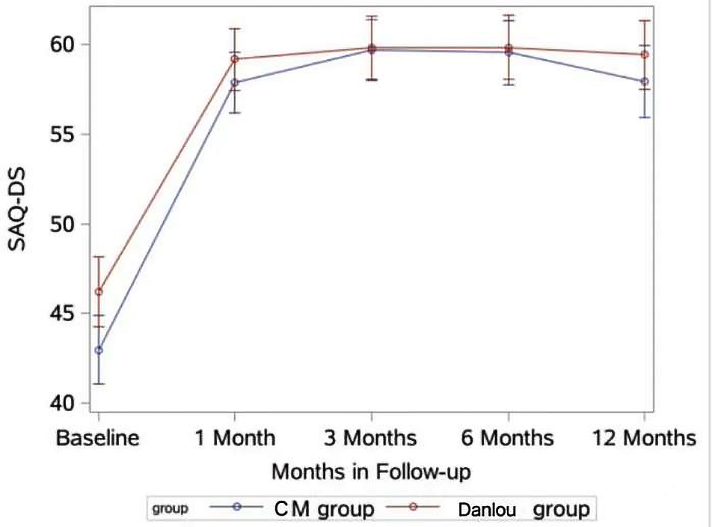

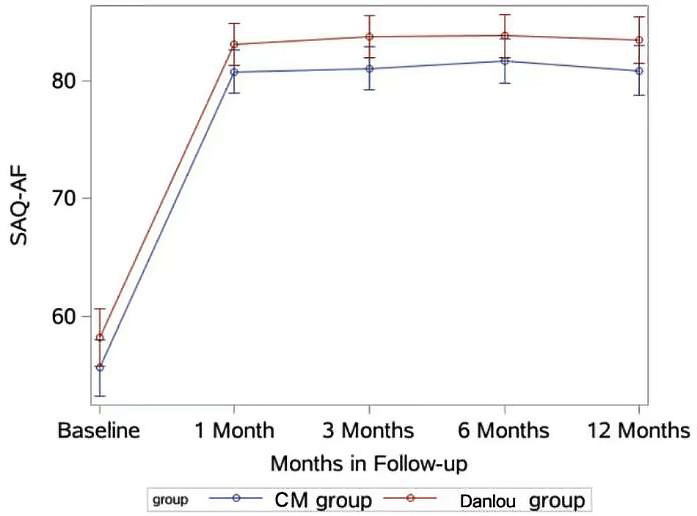

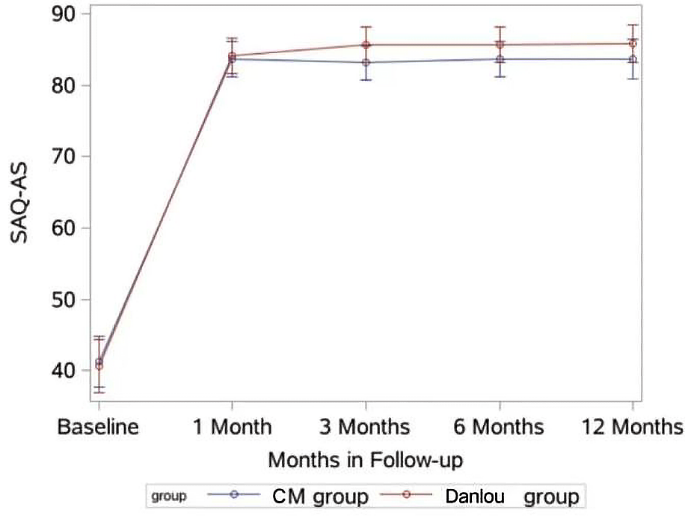

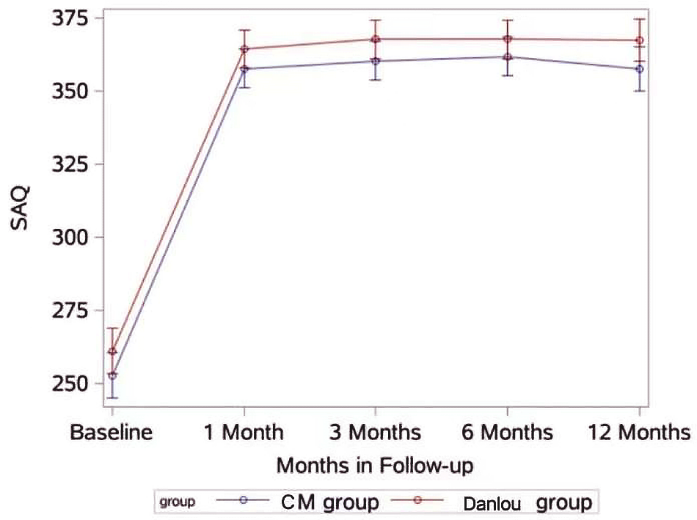

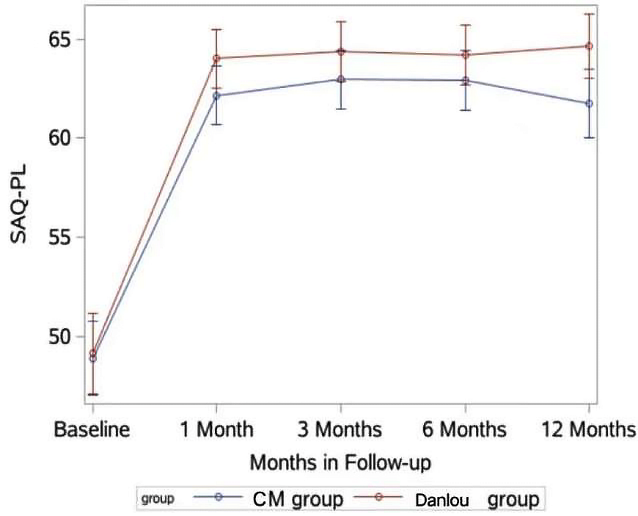


Figure S1. Comparison of trends in quality of life scores between groups. (SAQ and sub-domains of SAQ, DLT group=red, CM group=blue ). Abbreviation: SAQ: Summary score; SAQ_PL: Physical limitation; SAQ_AS: Angina stability; SAQ_AF: Angina frequency; SAQ_TS: Treatment satisfaction; SAQ_DS: Disease perception.

Table S2 Association between integrative group and Health Status by linear mixed effects models

| Outcomes | Univariate analysis | | | | Multivariable adjustment | | | | |
| --- | --- | --- | --- | --- | --- | --- | --- | --- | --- |
|  | β | 95%CI | | *P* value | β | | 95%CI | | *P* value |
| Summary score |  |  |  |  |  |  |  |  |  |
| Group | 14.49 | 6.01 | 22.97 | 0.001 | 13.19 | 4.03 | | 22.35 | 0.005 |
| Group-time interaction |  |  |  | 0.818 |  |  | |  | 0.813 |
| Physical limitation |  |  |  |  |  |  |  |  |  |
| Group | 3.20 | 1.19 | 5.22 | 0.002 | 2.94 | 0.75 | | 5.13 | 0.009 |
| Group-time interaction |  |  |  | 0.696 |  |  | |  | 0.696 |
| Angina stability |  |  |  |  |  |  |  |  |  |
| Group | 2.51 | -0.44 | 5.45 | 0.095 | 2.58 | -0.61 | | 5.78 | 0.113 |
| Group-time interaction |  |  |  | 0.036 |  |  | |  | 0.035 |
| Baseline | 6.27 | 2.51 | 10.04 | 0.001 | 6.34 | 2.38 | | 10.29 | 0.002 |
| 1-month | -4.38 | -8.17 | -0.59 | 0.023 | -4.38 | -8.17 | | -0.59 | 0.023 |
| 3-month | -5.43 | -9.27 | -1.59 | 0.006 | -5.45 | -9.29 | | -1.61 | 0.005 |
| 6-month | -5.19 | -9.08 | -1.3 | 0.009 | -5.2 | -9.09 | | -1.31 | 0.009 |
| 12-month | -4.51 | -8.66 | -0.37 | 0.033 | -4.54 | -8.68 | | -0.39 | 0.032 |
| Angina frequency |  |  |  |  |  |  | |  |  |
| Group | 2.41 | 0.03 | 4.80 | 0.047 | 2.25 | -0.33 | | 4.83 | 0.087 |
| Group-time interaction |  |  |  | 0.762 |  |  | |  | 0.761 |
| Treatment satisfaction |  |  |  |  |  |  |  |  |  |
| Group | 2.64 | 0.71 | 4.57 | 0.007 | 2.27 | 0.16 | | 4.38 | 0.035 |
| Group-time interaction |  |  |  | 0.416 |  |  | |  | 0.418 |
| Disease perception |  |  |  |  |  |  |  |  |  |
| Group | 3.51 | 1.29 | 5.74 | 0.002 | 2.93 | 0.51 | | 5.36 | 0.018 |
| Group-time interaction |  |  |  | 0.592 |  |  | |  | 0.592 |

Abbreviation: SD, standard deviation

Independent variable including treatment and time points in univariate analysis. Multivariable adjustment plus for family history, currently smoking, resting heart rate, systolic blood pressure, diastolic blood pressure, comorbidities (hypertension, hyperlipidemia, diabetes, stroke), concomitant medication (ACEIs, ARBs, beta-blockers, CCBs, statins), diagnosis of ACS (STE-ACS and NSTE-ACS), number of diseased vessels, diseased coronary artery (LM, LAD, LCX, RCA). P-value refers to comparison between subjects with DLT group and CM group.

Table S3 Frequencies of adverse events [n (%)] after treatment in patients with ACS undergoing PCI

| Adverse Events | DLT Group (n=443), (n/%) | CM Group (n=432), (n/%) | P |
| --- | --- | --- | --- |
| total | 34（7.67） | 32（7.41） | 0.806 |
| elevated ALT | 4（0.90） | 5（1.16） | 0.739 |
| elevated UA | 10（2.26） | 7（1.62） | 0.467 |
| diarrhea | 12（2.71） | 10（2.31） | 0.670 |
| vomiting | 4（0.90） | 7（1.62） | 0..366 |
| fever | 4（0.90） | 3（0.69） | 0.705 |

Abbreviation: ALT, alanine aminotransferase; UA, uric acid; CM, conventional medicine; DLT, Danlou Tablet. P-value refers to comparison between subjects with DLT group and CM group

Table S4 List of participating medical centers and principal investigators

| Centers | PIs |
| --- | --- |
| Xiyuan Hospital of China Academy of Chinese Medical Sciences | Dazhuo Shi |
| Beijing Anzhen Hospital, Capital Medical University | Shuzheng Lv |
| Central Hospital of Shijiazhuang | Chao Wang |
| Dezhou Hospital of Traditional Chinese Medicine | Hongxing Zhang |
| Dongzhimen Hospital,Beijing University of Chinese Medicine | Lijing Zhang |
| First Teaching Hospital of Tianjin University of Traditional Chinese Medicine | Jingyuan Mao |
| Foshan Foxing Chancheng Hospital | Yonghua Qiu |
| Fujian People's Hospital | Shangquan Xiong |
| Fuwai Hospital, Chinese Academy of Medical Sciences | Shijie You |
| Guangdong Hospital of Traditional Chinese Medicine | Huanlin Wu |
| Haici Medical Center of Qingdao | Wenyan Ji |
| Hospital Affiliated to Shandong University of Traditional Chinese Medicine | Feng Lu |
| Jiangsu Province Hospital of Chinese Medicine | Xiaohu Chen |
| Jilin Hospital of Traditional Chinese Medicine | Liping Chang |
| Jilin Province People's Hospital | Heping Liu |
| Longhua Hospital, Shanghai University of Chinese Medicine | Nuo Tang |
| Rocket Force General Hospital of PLA | Rong Zhang |
| Shanghai Pudong Gongli Hospital | Lirong Wang |
| Shanghai Seventh People's Hospital | Changzhu Zheng |
| Shuguang Hospital, Shanghai University of Chinese Medicine | Xiaolong Wang |
| Taian Hospital of Traditional Chinese Medicine | Lin Zhang |
| The 464th Hospital of the PLA | Shenghua Ding |
| The Affiliated Hospital of Qingdao University | Wei Guo |
| The Eight Medical center of PLA generral Hospital | Lejian Lin |
| The First Affiliated Hospital of Henan University of Traditional Chinese Medicine | Mingjun Zhu |
| The First Hospital of Fangshan District,Beijing | Xuemei Peng |
| The Third Hospital of Hebei Medical University | Wenliang Xiao |
| The Third Hospital of Shijiazhuang | Ping Jiang |
| Tianjin Hospital of Nankai Intergrative Medicine | Qun Dang |
| Tianjin Chest Hospital | Chunjie Li |
| Tianjin First Central Hospital | Chao Wang |
| Traditional Chinese Medical of Xinjiang Uygur Autonomous Region | Xiaofeng Wang |
| Wuxi Traditional Chinese Medicine Hospital | Shu Lu |
| Xiamen Hospital of Traditional Chinese Medicine | Changyi Guan |
| Xuzhou City Hospital of Traditional Chinese Medicine | Zhongliang Wang |
| Yanbian University Hospital | Yuzi Li |
| Yantai Affiliated Hospital of Binzhou Medical University | Mengsong Shi |
| Yueyang Hospital of Integrated Traditional Chinese and Western Medicine，Shanghai University of Traditional Chinese Medicine | Haiming Luo |
| Zhengzhou Central Hospital | Shouyan Zhang |
| ZiBo Central Hospital | Bo Yin |

**1. Detailed definitions of primary and secondary endpoint events.**

The primary endpoint was a composite outcome that included cardiac death, nonfatal myocardial infarction, and urgent revascularization. The secondary endpoint encompassed rehospitalization owing to ACS, heart failure, stroke, and other thrombotic events such as pulmonary embolism, intermittent claudication, and deep venous thrombosis of the lower extremities.

**Endpoint events are defined in detail below:**

**Cardiac death**, including death caused by malignant arrhythmias, acute myocardial infarction, heart failure, cardiogenic shock, and other cardiovascular causes.

**Criteria for acute myocardial infarction (1)**

Detection of a rise and/or fall in cardiac biomarker values (preferably cardiac troponin [cTn]) with at least one value above the 99th percentile upper reference limit (URL) and with at least one of the following :

**Symptoms of ischemia:**

New or presumed new significant ST-segment–T wave (ST–T) changes or new left bundle branch block (LBBB).

Development of pathological Q waves in the ECG.

Imaging evidence of new loss of viable myocardium or new regional wall motion abnormality.

Identification of an intracoronary thrombus by angiography or autopsy.

Stent thrombosis associated with MI when detected by coronary angiography in the setting of myocardial ischemia and with a rise and/or fall of cardiac biomarker values with at least one value above the 99th percentile URL.

Classification of STEMI and NSTEMI based on ST segment elevation or depression:

ST elevation—New ST elevation at the J point in two contiguous leads with the cut-points: ≥0.1 mV in all leads other than leads V2–V3 where the following cut points apply: ≥0.2 mV in men ≥40 years; ≥0.25 mV in men <40 years, or ≥0.15 mV in women.

ST depression and T-wave changes—New horizontal or down-sloping ST depression ≥0.05 mV in two contiguous leads and/or T inversion (≥0.1 mV) in two contiguous leads with prominent R wave or R/S ratio >1.

**Repeat urgent revascularization:** The patient required urgent admission for revascularization due to aggravated angina, new ischemic changes on electrocardiogram, or elevated biomarkers.

**Rehospitalization due to ACS**: rehospitalization due to UA, STEMI, or NSTEMI.

**Heart failure:**

**Diagnosis of heart failure and reduced ejection fraction (2)**: Typical symptoms such as breathlessness, ankle swelling, and fatigue; typical signs typical for instance elevated jugular venous pressure, pulmonary crackles, and displaced apex beat; reduced left ventricular ejection fraction (EF≤40%).

**Diagnosis of heart failure with preserved ejection fraction:** Typical symptoms and signs; normal or mildly reduced left ventricular ejection fraction and left ventricular not dilated (40% < EF < 50%); relevant structural heart disease (left ventricular hypertrophy/left atrial enlargement) and/or diastolic dysfunction.

**Stroke:** a neurological defect caused by an ischemic or hemorrhagic central nervous system event. Neurological symptoms or signs last at least 24 hours after onset and may even lead to death.

**Other thrombotic events,** including pulmonary embolism, intermittent claudication, and deep vein thrombosis of the lower extremities.

**References**

[1] Thygesen K, Alpert JS, Jaffe AS, Simoons ML, Chaitman BR, White HD, et al. Third universal definition of myocardial infarction. J Am Coll Cardiol (2012) 60:1581–98. doi: [10.1016/j.jacc.2012.08.001](https://doi.org/10.1016/j.jacc.2012.08.001).

[2] Mant J, Al-Mohammad A, Swain S, Laramée P, Guideline Development Group. Management of chronic heart failure in adults: Synopsis of the National Institute for Health and Clinical Excellence guideline. Ann Intern Med (2011) 155:252–9. doi: [10.7326/0003-4819-155-4-201108160-00009](https://doi.org/10.7326/0003-4819-155-4-201108160-00009).

2. **Sample Size Calculation**

The sample size for this study was calculated using PASS 11. The primary endpoint was the incidence of adverse cardiovascular events. Based on literature reports[1], it is assumed that the incidence of adverse cardiovascular events in the treatment group will be 22.0% post-treatment, compared to 33.6% in the control group post-treatment. With a power of 80% and a false-positive error rate controlled at 5% (two-sided), and an allocation ratio of 1:1 between the two groups, 466 patients need to be enrolled (233 in the treatment group and 233 in the control group). Considering a 20% dropout rate during follow-up, the final sample size is determined to be at least 518 patients (259 in the treatment group and 259 in the control group)

References:

[1]Wang L, Zhao X, Mao S, Liu S, Guo X, Guo L, et al. Efficacy of Danlou tablet in patients with non-ST elevation acute coronary syndrome undergoing percutaneous coronary intervention: Results from a multicentre, placebo-controlled, randomized trial. Evid Based Complement Alternat Med (2016) 2016:7960503. doi: 10.1155/2016/7960503
